# Examining Historical and Current Mixed-Severity Fire Regimes in Ponderosa Pine and Mixed-Conifer Forests of Western North America

**DOI:** 10.1371/journal.pone.0087852

**Published:** 2014-02-03

**Authors:** Dennis C. Odion, Chad T. Hanson, André Arsenault, William L. Baker, Dominick A. DellaSala, Richard L. Hutto, Walt Klenner, Max A. Moritz, Rosemary L. Sherriff, Thomas T. Veblen, Mark A. Williams

**Affiliations:** 1 Earth Research Institute, University of California Santa Barbara, Santa Barbara, California, United States of America; 2 Environmental Studies Department, Southern Oregon University, Ashland, Oregon, United States of America; 3 Earth Island Institute, Berkeley, California, United States of America; 4 Canadian Forest Service Natural Resources Canada, Corner Brook, N.L., Canada; 5 Program in Ecology and Department of Geography, University of Wyoming, Laramie, Wyoming, United States of America; 6 Geos Institute, Ashland, Oregon, United States of America; 7 Avian Science Center, Division of Biological Sciences, University of Montana, Missoula, Montana, United States of America; 8 Wildlife Habitat Ecologist, FLNR, Thompson-Okanagan Region, Kamloops, B.C., Canada; 9 Ecosystem Sciences Division, Environmental Science, Policy, & Management Dept., University of California, Berkeley, California, United States of America; 10 Department of Geography, Humboldt State University, Arcata, California, United States of America; 11 Department of Geography, University of Colorado Boulder, Boulder, Colorado, United States of America; 12 Program in Ecology and Department of Geography, University of Wyoming, Laramie, Wyoming, United States of America; University of New South Wales, Australia

## Abstract

There is widespread concern that fire exclusion has led to an unprecedented threat of uncharacteristically severe fires in ponderosa pine (*Pinus ponderosa* Dougl. ex. Laws) and mixed-conifer forests of western North America. These extensive montane forests are considered to be adapted to a low/moderate-severity fire regime that maintained stands of relatively old trees. However, there is increasing recognition from landscape-scale assessments that, prior to any significant effects of fire exclusion, fires and forest structure were more variable in these forests. Biota in these forests are also dependent on the resources made available by higher-severity fire. A better understanding of historical fire regimes in the ponderosa pine and mixed-conifer forests of western North America is therefore needed to define reference conditions and help maintain characteristic ecological diversity of these systems. We compiled landscape-scale evidence of historical fire severity patterns in the ponderosa pine and mixed-conifer forests from published literature sources and stand ages available from the Forest Inventory and Analysis program in the USA. The consensus from this evidence is that the traditional reference conditions of low-severity fire regimes are inaccurate for most forests of western North America. Instead, most forests appear to have been characterized by mixed-severity fire that included ecologically significant amounts of weather-driven, high-severity fire. Diverse forests in different stages of succession, with a high proportion in relatively young stages, occurred prior to fire exclusion. Over the past century, successional diversity created by fire decreased. Our findings suggest that ecological management goals that incorporate successional diversity created by fire may support characteristic biodiversity, whereas current attempts to “restore” forests to open, low-severity fire conditions may not align with historical reference conditions in most ponderosa pine and mixed-conifer forests of western North America.

## Introduction

In just two days in 1910, 1.2 million ha of forestlands in Idaho and Montana in the western USA burned in a massive fire driven by exceptional winds [1]. In the aftermath, the United States instituted a policy of aggressive fire suppression [2]. Decades of fire suppression activities since 1910 have reduced the extent and number of wildfires in the USA, as well as parts of Canada. There is now widespread concern that fire exclusion has caused vegetation in western North America to be much more susceptible to uncharacteristically severe fire. This concern is greatest in the extensive, often drier forests of the North American Cordillera, especially those dominated by ponderosa pine (*Pinus ponderosa* Dougl. ex. Laws) and Jeffrey pine (*P. jeffreyi* Grev. & Balf.), or those mixed with ponderosa/Jeffrey-pine and other conifer species (hereafter ponderosa pine and mixed-conifer forests of western North America, defined in Table 1 and further described in Methods).

**Table 1:** Definitions of terms as used in this paper.

Term	Definition
Ponderosa pine andmixed-conifer forests ofWestern North America	Low- to mid-elevation, montane, non-coastal forests of western North America where a regime of low/moderate-severity fire (see Table 2 for explanation) that limit tree recruitment has traditionally been applied. These extensive forests are dominated by ponderosa pine (*Pinus ponderosa*), Douglas-fir (*Pseudotsuga menziesii*) and fir (*Abies concolor* and *A. grandis*) (see Methods). These forests are drier than coastal forests or most forests at higher elevations, though one region, the Klamath, is more mesic.
Fire dependent	Biota that occur most abundantly after high-severity fire, and which are largely or entirely absent where high-severity fire has not occurred for a long period.
Fire regime	The frequency, size, seasonality, impacts and other characteristics of naturally occurring fires that have occurred in a vegetation type over its lifespan, generally 1–3 millennia [133].
High-severity fire rotation (or moderate to high-severity fire rotation)	The length of time required for an area equal to the area of interest to burn [134]. For high-severity fire, this is calculated as the time period over which high-severity fire (or moderate- and high severity fire combined) is observed, divided by the proportion of the area of interest that burns in that time period at high- or moderate/high-severity.
High-severity fire	Fire that burns on the ground surface, and typically in the overstory canopy (crown fire) as well. Mortality of woody species as measured by basal area is generally >70%. However, sprouting canopy species, such as oaks (*Quercus* spp.) typically survive these fires. High-severity fire mainly occurs in relatively discrete patches under high winds that cause blow ups in fire behavior [108]. These patches range in size from the area occupied by a small group of trees to many thousands of ha in size, as in the case of the 1910 fires.
Low-severity fire	Fire that burns on the ground surface such that relatively little or no mortality of live, standing vegetation occurs. Mortality of woody species as measured by basal area is 0–20%, but is mostly 0–5%. See Table 2 for a detailed explanation of the effects of a regime of low-severity fire.
Moderate-severity fire	Fire that burns only on the ground surface and that has effects that are intermediate between low- and high-severity fire as defined here. Mortality of woody species as measured by basal area is generally 20–70% within a given area.
Mixed-severity fire	Fire that includes low-, moderate-, and high-severity effects. See Table 2 for a detailed explanation of the effects of a regime of mixed-severity fire.
Park-like forest	A forest of widely-spaced live, mature trees and very few, if any, dead trees (snags). The understory is open, often dominated by bunchgrasses, and is mostly lacking woody plants.
Stand age	The age within a stand of the dominant overstory canopy vegetation that recruited more or less as a cohort, typically after a previous disturbance.

These terms may have different meanings in the literature depending on the context in which they are used.

The ponderosa pine and mixed-conifer forests of western North America have traditionally been considered adapted to a low- or low/moderate-severity fire regime (see Tables 1 and 2 for definitions of fire terms) [3]–[8]. There have been many large mixed-severity fires in western North America in recent years [9] that have helped create widespread concern that fire exclusion has caused an unprecedented threat of uncharacteristically severe fires [6]–[15]. Concomitantly, however, there has been increasing recognition that fires in ponderosa pine and mixed-conifer forests of western North America were also mixed in severity prior to any significant effects of fire exclusion (Table 2) [16], [17]. It has also been increasingly recognized that these forests support biota that are not adapted to low/moderate-severity fire, but rather are dependent on the high-severity fire component of mixed-severity regimes [18]–[22]. Thus, a better understanding of historical (i.e., generally prior to fire suppression and timber harvesting) fire regimes in these forests is needed to define reference conditions and maintain characteristic ecological diversity.

**Table 2:** Characteristics of fire regimes in ponderosa pine and mixed-conifer forests of Western North America.

	Low/moderate-severity model	Mixed-severity model
Tree populations	1. Stable. Gap phase recruitment dynamics.	1. Unstable. Gap and stand-level mortality and recruitment. Stand-replacement fires at intervals often shorter than tree lifespans.
	2. Recruitment limited by frequent fire.	2. Recruitment abundant and stimulated by fire.
	3. Resistant to fire (though oftendescribed as “fire-resilient”).	3. Resilient following fire.
Landscape patterns	1. Successional diversity low.	1. Successional diversity high.
	2. Gradual variation alongenvironmental gradients.	2. Variation along environmental gradients interrupted by sharp boundaries and patchiness.
	3. Low contrast heterogeneity.Intensity/complexity ofspatial pattern is low.	3. High contrast spatial heterogeneity. Intensity/complexity of spatial pattern is high.
	4. Low beta diversity.	4. High beta diversity.
Stand structure	1. Does not vary markedly over time.	1. Varies markedly as a function of time since fire disturbance.
	2. Open canopy of mature, medium andlarge trees; density low.	2. Variable canopy, tree size, and density variable; even-aged cohorts stimulated by fire.
	3. Understory with few trees or shrubs.	3. Understory varies.
Fire behavior	1. Typically low intensity surface firewith flame lengths <3 m;short residence time.	1. Variable intensity surface or crown fire, variable residence time.
	2. Fuel limited. Crown fire cannot initiate.	2. Not necessarily fuel limited. Crown fire can initiate under extreme conditions.
Individual firecanopy mortality	1. Mortality of canopy trees <20% by basal area.	1. Mix of low-, intermediate- and high-severity fire with (0–20%, 20–70%, >70%) mortality of canopy trees by basal area respectively.
Interactive effects of fire on fuels and forest flammability	1. Fires continuously limit fuels and fire sensitive trees.	1. Fires only temporarily lower fuels.
	2. Maintain low flammability and forestmortality over time.	2. Do not maintain low flammability and forest mortality, except initially after fires.
Evolutionary responses	1. Fire resistant trees.	1. Fire resistant and fire-dependent or specialized biota. The latter includes species with reproduction timed to coincide with fire via fire-cued germination, fire “embracer” plant species, and post-fire insect and bird specialists).
Fire exclusion leads to	1. High tree regeneration*.	1. Low tree regeneration.
	2. Greatly increased flammability.	2. Small changes in flammability (vegetation is continuously flammable except initially after fire).
	3. Increased forestsusceptibility to mixed-severity fire.	3. Decreased susceptibility to mixed-severity fire.
Carbon storage^1^	1. Low-moderate; considerably lower than carrying capacity.	1. Moderate to high; Near carrying capacity.
Fuel treatments (forest thinning)	1. Restores forest tree structure and fuelloads where infill associated withfire exclusion is removed.	1. May create uncharacteristic structure and composition (reduction in small and intermediate and some overstory trees, shrubs, down wood).
	2. Increase open forest (woodland) biota.	2. Decrease in dense forest biota and post-fire habitat specialists.
	3. May create low contrast heterogeneity.	3. May reduce high-contrast heterogeneity.

1 [135]–[137].

In recent decades, to address the widespread concerns about uncharacteristically severe fire in western North America, fuel reduction treatments have been implemented on millions of hectares of ponderosa pine and mixed-conifer forests at a cost of billions of dollars [23]. These treatments consist mainly of harvesting smaller trees to reduce forest density [8], but larger trees are typically harvested as well for economic reasons [24]. These treatments can negatively affect fire dependent species. For example, the Black-backed Woodpecker (*Picoides arcticus*), an imperiled fire-dependent species, largely avoids previously thinned forest areas burned at high-severity [18]. Thinning treatments also eliminate/degrade dense forest, which many species need, including the Northern Spotted Owl (*Strix occidentalis caurina*), a Threatened Species under the USA Endangered Species Act [25], and the Pacific fisher (*Pekania pennanti*), a Candidate Species under the USA Endangered Species Act [26]. In addition, forest thinning treatments often require the reopening or construction of access roads, which have many ecosystem impacts [27], and both the thinning treatments and roads promote the establishment of invasive species [27], [28]. Thinning ultimately exacerbates fire suppression impacts if it facilitates fire control, or if it becomes a prerequisite for allowing wildfires to burn [13], [29]. Thus, there is a need to ensure that actions are ecologically justified.

Most descriptions of the fire regimes that characterize the ponderosa pine and mixed-conifer forests of western North America (e.g., [5]–[7], [11]) emphasize how low-severity fires maintain forests dominated by relatively old and large, fire-resistant trees, with few understory trees, dead or dying trees, or shrubs [3]–[7], [11]–[13] (Table 2). Park-like conditions and low fuel loads are thought to result from effects of frequent surface fire, which kills young, fire-sensitive trees, while older, fire-resistant trees survive [4], [6], [7], [11], [12].

In contrast, mixed-severity fire regimes are characterized by more variable fire and forest structure across a wide range of spatial and temporal scales [17], [21] (Tables 1 and 2). The creation of complex early seral vegetation by high-severity fire often occurs in irregular patches across the landscape and at irregular intervals [30]. Over time, the complex early successional vegetation created by fire, if not reburned, transitions to mid- and then late-successional forest, often containing pre-disturbance legacies, such as standing or fallen dead trees and often some fire resistant, large trees that survive fire crown fire (e.g., [31]). Thus, mixed-severity fire regimes create complex successional diversity high beta diversity, and diverse stand-structure across the landscape [17], [21], [30], [32]–[35].

The concepts and nomenclature used to describe fire regimes in western North America can be ambiguous. Part of the problem with defining fire regimes for the drier forests of western North America is the classification of fire regimes into distinct categories of low-, mixed-, and high-severity [5], or low/moderate-severity and high-severity [9], when nearly all fire regimes include a mix of all three severities. Greater clarity in terminology is needed to improve communication about fire regimes. Tables 1 and 2 document the terminology used herein.

In addition to unclear terminology, other factors create difficulties for identifying which historical (i.e. prior to fire exclusion) fire regime applies to a particular forest region. Where fire has been excluded from a mixed-severity landscape for 100 years, early- and mid-successional patches created by high-severity fire become late-successional patches, making it more likely that these patches, indicative of a mixed–severity regime, will be undetected. For example, high-severity fire patches may be detected in old but not recent aerial imagery [35]. A primary source of data on historical fires are scars in the growth rings of surviving trees damaged by fire, which can provide annually precise dates for past fires at the sampled locations [36]–[40]. However, these methods cannot effectively determine past occurrence of high-severity fire. Thus, additional evidence is needed to characterize historical fire regimes over more extensive areas.

The US Forest Service Inventory and Analysis (FIA) program provides an extensive dataset that is a probabilistic sample of forest structure in large landscapes. This dataset allows for landscape-scale inference and statistical analyses of forest age and structure parameters consistent with a low- or mixed-severity fire regime.

Using the FIA data, and published sources of landscape-scale (area of inference >25,000 ha) data, our objectives were to address two broad questions: (1) How prevalent were mixed-severity fire regimes historically in ponderosa pine and mixed-conifer forests of western North America; and (2) How have mixed-severity fire patterns in these forests changed with fire exclusion? Consistent with common perceptions and restoration models applied to these forests, we hypothesized that: (1) forest age-class diversity was low, reflecting long-term effects of low/moderate-severity fire regimes (Table 1); and (2) fire exclusion has led to vegetation changes that have increased the prevalence of high-severity fire.

## Methods

### Study Area

**Figure 1:**
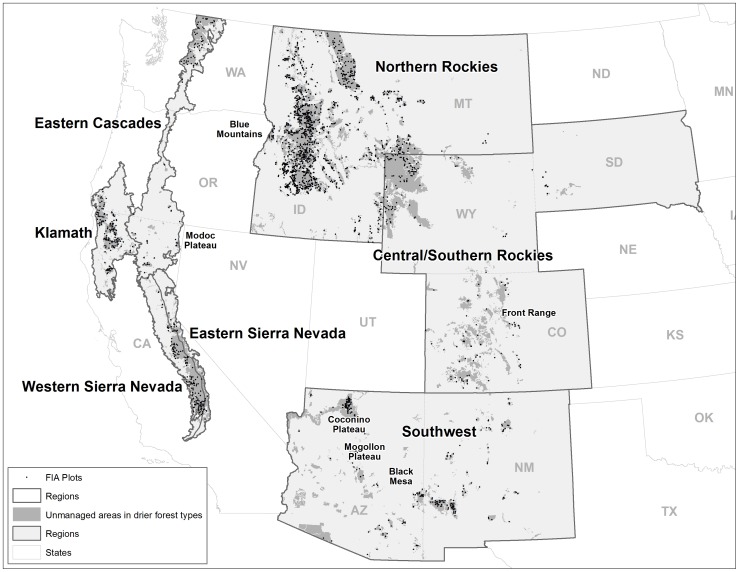
Study area. Dots indicate the general locations of Forest Inventory and Analysis (FIA) plots.

FIA and published sources of landscape-scale (area of inference >25,000 ha) data with inference to pre-settlement fire severity and forest structure were available from the following regions of western North America: Baja California, the Sierra Nevada, the Klamath Region, the eastern Cascades, the northern Rockies, the central Rockies, and the southwestern USA (Figure 1). We used ecoregional class III data from the US Environmental Protection Agency (http://www.epa.gov/wed/pages/ecoregions/level_iii_iv.htm) to define the Sierra Nevada, Klamath, and eastern Cascades regions. The Sierra Nevada was split along the distinct crest of the range into the east and west slopes. The portions of the northern Cascades east of the crest and the main Cascades within California were combined into the eastern Cascades. The Modoc Plateau and eastern Sierra Nevada was also combined with the eastern Cascades. The northern Rockies were in Idaho and Montana, and the central Rockies were in Colorado, Wyoming and South Dakota. The southwestern USA included Arizona and New Mexico.

The dominant conifer over most of the low- to mid-elevation, montane forests in these regions is ponderosa pine, often with lesser amounts of Douglas-fir, white fir (*Abies concolor* (Gord. and Glend.) Lindl.), and/or grand fir (*A. grandis* (Douglas ex D. Don) Lindl.). In the Sierra Nevada and Klamath regions, ponderosa pine is common and may be dominant, especially in low-elevation forests, and mixed-conifer forests generally include components of ponderosa pine, white fir, Douglas-fir, incense-cedar (*Calocedrus decurrens* (Torr.) Florin), sugar pine (*P. lambertiana* Dougl.), California black oak (*Quercus kelloggii* Newb.) and evergreen canyon live oak (*Q. chysolepsis* Liebm.). Mid-elevation forests of the Sierra Nevada and Cascades are often dominated by Jeffrey pine, ponderosa pine, white fir and sugar pine. Low- to mid-montane forests of the eastern Cascades are dominated by ponderosa pine and Douglas-fir, and can include components of white fir, grand fir (*Abies grandis* Dougl. ex D. Don.) Lindl.), and western hemlock (*Tsuga heterophylla* (Raf.) Sarg). Low- and mid-elevation forests of the Rocky Mountains are dominated by ponderosa pine and Douglas-fir. In the northern Rockies, these two dominants may co-occur with white fir and grand fir, and with western hemlock, western redcedar (*Thuja plicata* Donn. Ex D. Don.) and quaking aspen (*Populus tremuloides*). Forests of the southwestern U.S. are heavily dominated by ponderosa pine, with some white fir and Douglas-fir at middle elevations. Precipitation and temperature data for each region in this study are provided in Table 3.

**Table 3:** Mean annual precipitation, and mean summer maximum and minimum temperatures, in ponderosa pine and mixed-conifer forests in each region.

Region*	Mean annual precipitation(cm)	Mean maximum temperature,June-August (degrees C)	Mean minimum temperature,June-August (degrees C)
Sierra Nevada	104	23	9
Klamath	196	26	11
Eastern Cascades and Eastern Sierra Nevada	113	21	7
Northern Rockies	88	22	6
Central and Southern Rockies	71	22	6
Southwest	58	27	11

* All values are from PRISM data (http://www.prism.oregonstate.edu/normals/) in each 2 km^2^ PRISM pixel within which an FIA plot used in the study occurred.

### Evidence for Historical Mixed-severity Fire Regimes in Ponderosa Pine and Mixed-conifer Forests

#### Rotations of high- and moderate-to high-severity fire

We summarized rotations for high-severity fire from published studies with inference to large landscapes (>25,000 ha) in ponderosa pine and mixed-conifer forest landscapes of western NA over a period of 70 or more years. The high-severity fire rotation is equal to the average interval between high-severity fire across the affected landscape (Table 1). Additionally, we summarized other evidence regarding the occurrence of high-severity fire where rotations could not be calculated, but where landscape-scale inference regarding the relative importance of high-severity fire was presented, or where rotations could be calculated but landscapes were <25,000 ha or the time period was <70 years.

#### Dominant overstory tree age distributions

To assess successional patterns indicative of mixed- vs. low/moderate-severity fire regimes, we analyzed US Forest Service Inventory and Analysis (FIA) stand ages (data available at http://www.fia.fs.fed.us/tools-data/) by region. These data capture the average age of the trees dominating the canopy layer in forest stands (stand age, Table 1) that have been sampled probabilistically, with inference to more extensive landscapes. Because the dominant trees in ponderosa pine and mixed-conifer forests may be several centuries old in the absence of disturbance [e.g., 41,42], we reasoned that the age of relatively young and intermediate-aged stands (e.g. <200 years) reflects the time since a disturbance that shifted dominance from older to younger trees. The FIA database indicated that young stands (generally 0–30 years) were initiated by fire. To determine whether disturbances in other plots were caused by fire, we evaluated the effects of fire exclusion on rates of disturbance, as described below. It is not possible to specify the level of mortality that fire or other disturbances may have caused, but it is possible to determine the extent to which forests were dominated by older age classes, which would be consistent with low−/moderate severity fire, versus stands of more diverse age classes, consistent with mixed-severity fire.

FIA is a monitoring system based on one permanent, random 1-ha plot per ∼2400 ha across forested lands in the USA. For tree measurements, the plot area is sub-sampled with four circular plots of 0.1 ha for large trees and 0.017 ha for smaller trees nested within the larger tree plots. Diameter at breast height (dbh) and crown position of each tree and the ring count from cores of the dominant and co-dominant trees (i.e., the main overstory canopy layer) of each tree species are measured in each subplot [43]. The stand-age variable for a “stocked” FIA plot (i.e., one containing trees of any age) is determined from the average of all ring counts from subplot samples of dominant and codominant trees in the size class characteristic of the overstory canopy structure, weighted by cover of sampled trees, and 8 years are added for estimated time to grow to breast height (1.4 m) at which cores are sampled.

We selected FIA data from low- to mid-elevation forest types in Wilderness, Inventoried Roadless Areas, and National Parks to ensure as best we could that stand initiation was not caused by commercial harvesting of trees or other land use (Fig. 1). We had no independent way to confirm that trees were never cut at each plot location, so we interpret the results assuming only that such management was of minor importance, given that Wilderness, Roadless, and National Park designations reflect a lack of past timber harvesting. We selected lands classified as “timberlands” in Pacific states’ data sets. In the Rockies and southwestern USA, where there was no such designation, we selected all areas where the potential vegetation was considered capable of >10 percent tree cover.

A small number of plots had different stand ages for different subplots due to disturbances that affected some, but not all, subplots. In FIA split-age plots where both plot ages were ≤100 years, plots were split into two stand ages by FIA if they differed by as little as 1 year. In split-age plots in which both ages were 100–199 years old, plots were split into two stand ages if they differed by as little as 2 years. In split-age plots where both ages were ≥200 years, plots were split into two stand ages if they differed by as little as 15 years. To assess the within plot variability in tree ages, we calculated the standard deviation of the trees used to age each plot. We standardized this across the range of stand ages by calculating the standard deviation of the proportional difference between stand age, and the individual trees used to determine stand age in each plot, over the range of stand ages.

**Figure 2:**
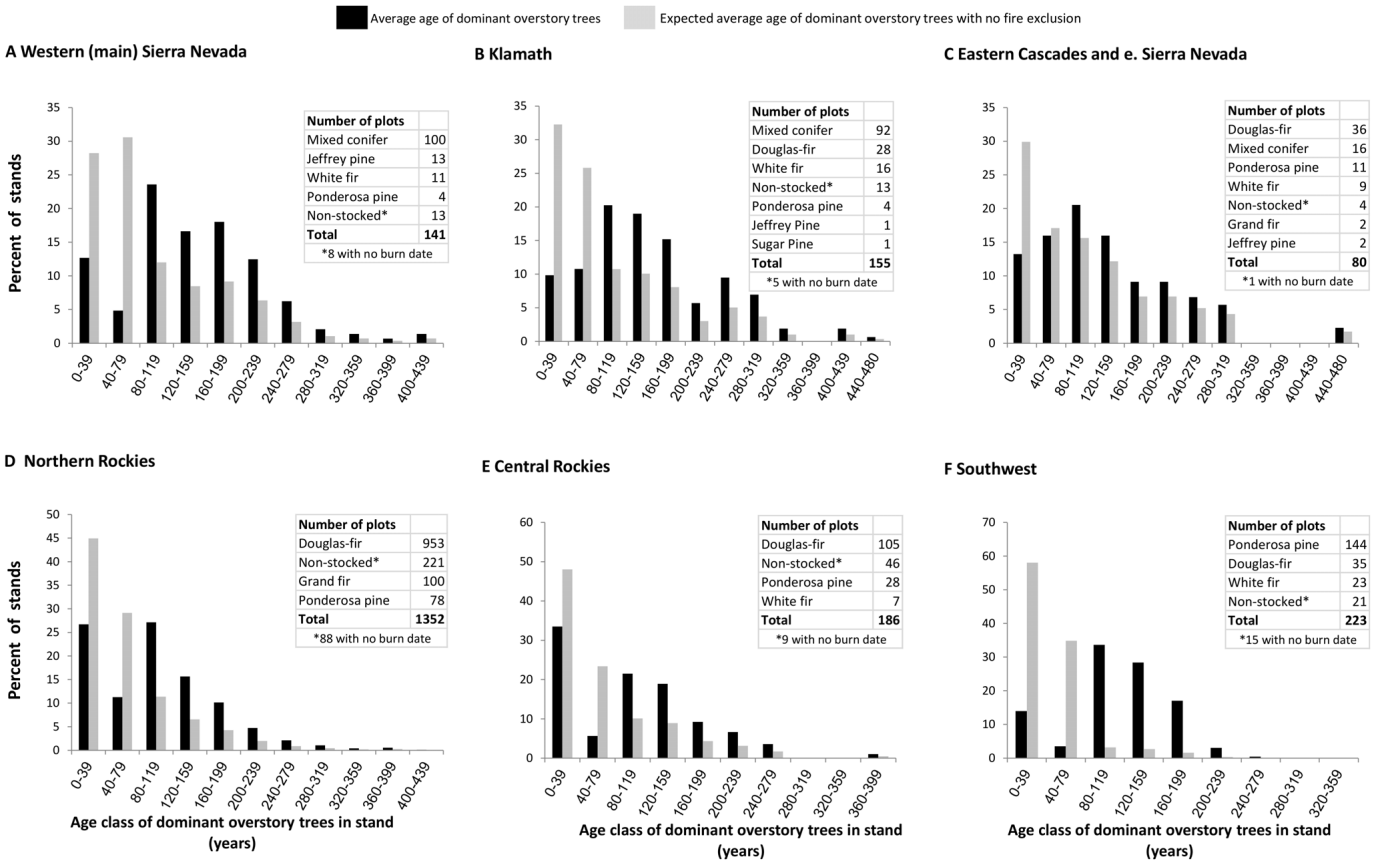
Age class distributions of dominant overstory trees. Data are from US Forest Service Forest Inventory and Monitoring plots from forested areas protected from logging in A. the western (main) Sierra Nevada, B. the Klamath Region, C. the eastern Cascades and Sierra Nevada, D. the northern Rockies, E. the central/southern Rockies, and F. the southwestern USA. Shown in black bars is the current distributions of stand ages. Grey bars show an expected distribution (average age of dominant overstory trees with no fire exclusion), based on projecting the occurrence of the same age distributions that occurred from 1810–1889 into the most recent 80 year time period and rescaling these data. The number of plots by forest type are shown in the imbedded tables. Non-stocked stands are those lacking trees that grew after the fire that could be aged non-destructively.

We reasoned that, prior to fire suppression, under a low/moderate-severity fire regime, successional, or age-class diversity, would be low, while it would be high under a mixed-severity fire regime. With fire exclusion and greater amounts of uncharacteristically severe fire the pattern should reverse in both cases (i.e. increased age-class diversity in low-severity systems and decreased diversity in mixed-severity systems). We used a Chi-square test of proportions [44] to test the null hypothesis that there would be no difference between the actual distribution of stand ages and the distribution based on a hypothetical scenario of no fire exclusion. No effect of fire exclusion would indicate that fire was not a dominant influence on age class diversity. To create a distribution of average dominant stand ages by region that would exist in today’s stands had fire exclusion never occurred, we used the distribution of plots with stand ages dating from 1889 or before. This time period was immediately prior to the onset of fire suppression activities by settlers and government agencies [35], [45]–[56]. Because the average tree ages are somewhat imprecise, we binned the data into 40-year age classes for hypothesis testing. In each region, the present age structure for 80 years during effective fire suppression (1930–2009) was compared with the age structure prior to fire suppression (1810–1889). For visual analysis, we shifted the pre-fire suppression (pre-1890) tree age distributions to present (i.e., shifting 1810–1849 to 1930–1969, and shifting 1850–1889 to 1970–2009) to compare with the current age distributions (see Figure 2). This allows a clear, visual comparison of stand ages that currently exist with those that would exist had the same fire regime from 1810–1889 occurred from 1930–2009.

We included only plots where there was one stand age for the full plot because we wanted to evaluate high-severity fire occurrence in patches at least 1 ha in size, rather than include smaller torching of groups of trees. Excluding the split-age plots (27% of plots in the Sierra Nevada, 40% in eastern-Cascades/eastern-Sierra, 26% in Klamath, 14% in northern Rockies, 36% in central/southern Rockies and 14% in southwestern USA) omits some additional evidence for local high-severity fire effects; thus our results may be conservative.

We used FIA data drawn from 2001–2009, comprising 90% of available plots, in our classification of low/mid-elevation forests in the Sierra Nevada, Klamath, and eastern Cascades. In the other regions, FIA plots represented 100% of the data from low- to mid-elevation, montane forests. The number of plots in the 0–39 year age bins may be slight underestimates of the amount of high-severity fire in the last 40 years because severe fire could have occurred subsequent to the sample date (plots were sampled between 1995–2009 in the northern, central and southern Rockies, and southwestern USA and 2001–2009 in the Sierra Nevada, Klamath and eastern Cascades). To estimate the number of plots that burned severely after the sample date, we increased the 0–39 year old bin by a factor of 40/36 in the Sierra Nevada, Klamath and eastern Cascades, 40/34 in the northern Rockies and 40/32.5 in the central/southern Rockies and southwestern USA region. The denominator in these weightings is based on 40 minus the mean amount of time in which plots in each region could have burned after being sampled.

We used year of the recent fire disturbance, captured in the disturbance data field, to define the age of very young FIA plots not containing trees that could be aged in a non-destructive way (FIA surveys do not allow trees to be killed). These “non-stocked” plots were relatively rare, as reported in the results, and ages, based on fire dates, all fell within the 0–39 age category. Some non-stocked stands had no disturbance coded. In California, Oregon, and Washington (Pacific states), disturbances were only coded if they were <6 years old. We placed all non-stocked plots where no disturbance was coded in the database into the 0–39 stand age bin.

Next, we considered whether the age distributions as shaped by fire were consistent with mixed- or low/moderate-severity fire regimes. We reasoned that a wide range in the plot stand ages in a landscape would be consistent with age-class diversity created by mixed-severity fire, while stand ages that were evenly distributed in predominantly older age classes would be consistent with a low/moderate-severity fire regime. To test whether stand age distributions were consistent with mixed- or low/moderate-severity fire regimes, we again used a Chi-square comparison of proportions [44]. Specifically, we tested the probability that the actual age distributions differed from an expected stand-age distribution for a low/moderate-severity fire regime. The low/moderate-severity (expected) distribution was based on 12.5% of stands falling into each 40-year age class between 80–399 years (0–319) years at the onset of fire exclusion. Our null hypothesis was that there would be no significant difference between the actual and expected (low/moderate-severity) distributions.

Third, we tested, again using a Chi-square comparison of proportions [44], the hypothesis that there would be less evidence for historical mixed-severity fire in the generally drier ponderosa and Jeffrey pine stands than in the mixed-conifer forests (i.e., the pine forests would be more frequently dominated by older stands).

## Results

### Evidence for Historical Mixed-severity Fire Regimes in Ponderosa Pine and Mixed-conifer Forests

#### Rotations of high- and moderate- to high-severity fire

The studies that allow calculation of rotations of high-severity fire over large, ponderosa pine and mixed-conifer forest landscapes of western North America over time periods of at least 70 years include areas ranging from 40,700 to 1,193,200 ha (Table 4). These large landscapes totaled 2.2 million ha in Baja California, the Sierra Nevada, eastern Cascades, northern Rockies (Blue Mountains of Oregon), the Colorado Front Range and Arizona (Black Mesa and the Mogollon Plateau). Most of the evidence presented in these studies was from ponderosa pine forests.

**Table 4:** Rotations for high-severity and moderate-severity fire in low/mid-elevation conifer forests of western North America.

Region	Location	Source	Analysis area (ha)	Forest types	Tree mortality	Time period	Approximate rotation (years)
Pacific states	Northern Baja California	[118] 1 analysis of aerial photos	40,700	Mixed conifer and Jeffrey pine	>90% overstory mortality	1925–1991	300
	Northern Sierra Nevada	[51] 2 Ground surveys and detailedmaps	146,917	Mixed conifer, dominatedby ponderosa pine	75% mortality by volumewas mapped for patches>32.4 ha	1800–1900	488
	EasternCascades (Washington)	[17], [35] 3, 4Analysis of historical aerialphotos	175,200	Mixed conifer andponderosa pine	>70% tree mortality^6^	∼1830–1930	379–505
							
					>20% tree mortality^6^	∼1830–1930	115–128
	Eastern Cascades (Oregon)	[56] 5 Analysis of General Land Officesurvey data	123,500	Ponderosa pine	>70% tree mortality^7^	∼1768–1882	705
			140,400	Dry mixed conifer	>70% tree mortality^7^	∼1768–1882	496
Northern Rockies	Oregon (Blue Mountains)	[57] 5 Analysis of General Land Officesurvey data	304,700	Ponderosa pine forests	>70% tree mortality^7^	∼1740–1880	849
	Oregon (Blue Mountains)	[57] 5 Analysis of General Land Officesurvey data	304,700	Ponderosa pine forests	Moderate- and high-severity fire	∼1740–1880	235
Central Rockies	Central (Colorado Front Range)	[57], [73] 5 Analysis of GeneralLand Office survey data	65,500	Ponderosa pine forests	>70% tree mortality^7^	∼1705–1880	271
	Central (Colorado Front Range)	[58] 8 Analysis of General Land Officesurvey data	624,156	Mostly ponderosa pineand Douglas-fir	Moderate and high-severity fire	1809–1883	249
Southwest (Arizona)	Black Mesa	[57] 5 Analysis of General Land Officesurvey data	151,100	Ponderosa pine forests	>70% tree mortality^7^	∼1760–1880	217
	Mogollon Plateau	[57] 5 Analysis of General Land Officesurvey data	452,100	Ponderosa pine forests	>70% tree mortality^7^	∼1760–1880	828
	Mogollon Plateau	[57] 5 Analysis of General Land Officesurvey data	452,100	Ponderosa pine forests	Moderate- and high-severity fire	∼1760–1880	319
	Black Mesaand Mogollon combined	[57] 5 Analysis of General Land Officesurvey data	603,200	Ponderosa pine forests	>70% tree mortality^7^	∼1760–1880	522

Data from General Land Office or mapped data over large areas (>25,000 ha) over >70 or more years prior to fire exclusion.

1 Study area was dominated by mixed conifer and Jeffrey pine and minimally logged. Fire exclusion only began in the 1970s and has had only a modest impact [138]. Thus, historical and current rates are assumed to be comparable.

2 Analysis of Leiberg’s mapping of high-severity fire areas within unlogged mixed-conifer Sierran stands is found in [139]. According to Leiberg [51], most such fire occurred prior to 1850. In addition, he stated “If the many small lots [<32 ha] scattered throughout still growing stands were taken into account, the figure [amount of area burned severely] would be considerably increased.”

3 The numerator was estimated at 100 years based on ponderosa pine in this region [140], whose growth would surpass 30 cm dbh, rendering mixed and high-severity effects indistinguishable (see [35: Table 1]). This calculation is conservative because tree growth to 30 cm dbh in moister forests is faster than 100 years.

4 High- and mixed-severity fire consistent with a definition of >70% and 20–70% basal area mortality, respectively, was identified from overstory canopy percentage, the overstory size class, the understory size class, and the fire tolerance of the cover type (see [35: Table 1]). Large patch sizes of historical high severity fire (100s to >5000 ha) from this work are reported in [17].

5 The estimate is from the span of years over which fire effects were distinguishable, using forest structure evident in the Government Land Office historical survey data, divided by the fraction of the forested landscape in which those fires occurred [56]. Rotations for high-severity fire are determined by dividing the observation period (the period of time over which fire effects are distinguishable by stand structure) by the percentage of the landscape experiencing high-severity fire. The methods were found to have 14.4–23% accuracy compared to plot sampling.

6 High- and mixed-severity fire, consistent with a definition of >70% and 20–70%, respectively, were identified from overstory canopy percentage, the overstory size class, the understory size class, and the fire tolerance of the cover type (see [35: Table 1]).

7 High-severity consistent with a definition of >70% basal area mortality [35] was identified having a percentage of small trees >50% and a percentage of large trees <20% [56], [57], [73],

8 Estimated from the length of General Land Office section lines intercepted by moderate- and high-severity fire. Accuracy tests using the length of section lines intercepted by modern moderate- and high-severity fire yielded a relative error of 15.6%.

The high-severity fire rotations in Table 4 do not support the hypothesis that low/moderate-severity fire regimes were predominant in the majority of ponderosa pine and mixed-conifer forests of western North America. In all the large, forest landscapes for which data covering at least 70 years exist, high-severity fire rotations ranged from about 217 to 849 years [57], and were mostly ∼200–500 years. This is generally less than potential tree lifespans. For combined moderate- and high-severity fires in the eastern Cascades, rotations were 115–128 years (Table 4: [35]), while they were 249 years in the Colorado Front Range (Table 4: [58]). In the Blue Mountains (northern Rockies) and on the Mogollon Plateau in Arizona, high-severity fire rotations of 849 and 828 years, and moderate/high-severity fire rotations of 235 and 319 years, respectively [57], occurred. Where high-severity rotations are relatively long, as they are in these regions, forest structure in portions of the landscape will lack evidence for high-severity fire even though it occurs often enough to create age-class diversity. Thus, while about 40% of the Blue Mountains forests and about 62% of those on the Mogollon Plateau had evidence from GLO surveys of forests shaped by low/moderate-severity fire only [57], similar to the nearby Coconino Plateau [59], structural diversity created by high-severity fire was evident on the remainder of the landscape [57], [59].

Numerous other studies that describe historical patterns of fire behavior also have documented or described evidence for mixed-severity fire effects in the ponderosa pine and mixed-conifer forests of western North American, including the occurrence of large high-severity fire patches (Table S1), and high-severity fire occurring over substantial areas of smaller landscapes over a time period of only a few decades prior to fire exclusion (e.g., Klamath region and a transitional area between the Sierra Nevada and eastern Cascades [60]–[63]).

Previous studies (Table 4) have used evidence of past fire severity from a variety of sources: GLO and other survey data, historical aerial photos; and mapping of vegetation and burns done prior to fire exclusion. The GLO analyses have been formally assessed for accuracy [64]. The methods performed well for addressing general hypotheses about the presence or absence of vegetation shaped by low- or high-severity fire. This was tested using existing vegetation plot data with an error of 14.4–23% [64].

#### Plot age distributions

A total of 2119 FIA plots representing a sample population of about 5.1 million ha of unmanaged low- to mid-elevation, montane forests in six regions (Figure 1, Table 5) were included in our analysis. Stand ages from ponderosa pine and mixed-conifer forests across the western USA never managed for timber cover areas ranging from 192,200 ha in eastern Cascades-eastern Sierra Nevada to 3,244,800 ha in the northern Rockies. Average stand ages ranged from 0 to 814 years, with the oldest stand in ponderosa pine in the eastern Cascades. The within plot standard deviation of the proportional difference among individual tree ages and stand age across all plots was 0.14 (e.g., for stands 100 years old, one standard deviation would include individual trees ∼86–114 years old, and two standard deviations would include trees ∼72–128 years old).

**Table 5:** Forest Inventory and Analysis (FIA) data.

Region	Number of plots (n) andforest area randomlysampled (ha)	Mean FIA stand age (yrs)		Test for difference in stand initiation since 1930 vs. 1800–1900: Chi-square, *P*
		Current	In 1930	
Sierra Nevada (main)	n = 232 338,400	148	97	86.3, <<0.001
E. Cascades and E. Sierra Nevada	n = 135 192,000	155	114	25.4, <<0.001
Klamath Mountains	n = 251 372,000	157	111	43.9, <<0.001
Central Rockies	n = 276 446,400	105	75	58.9, <<0.001
Northern Rockies	n = 1929 3,244,800	105	70	333.8<<0.001
Southwestern US	n = 319 492,000	116	59	188.2<<0.001

Area of sample population randomly sampled, mean stand age currently, and in 1930, and Chi-square test results.

The comparison of actual stand ages from 1930–2009 and the rescaled (expected) stand ages from 1810–1889 assuming no effect of fire exclusion are shown in Figs. 2A–F. In all regions, there were highly significant differences between the actual and expected stand age distributions (average ages of dominant trees with no fire exclusion) (*P*<0.001, Fig. 2A–F), indicating that fire was the predominant disturbance prior to effective fire exclusion. The FIA database also indicates that, since the onset of fire suppression, the great majority of stands were initiated by fire. As illustrated by the abundance of plots with stand ages that date to the decades prior to fire exclusion (e.g. 80–160 years old presently), much of the landscapes had young forests, but the rate of establishment decreased dramatically after 1930 (stand ages <80 years are rare). The rate of young forest establishment decreased by a factor of 4 in the Sierra Nevada and southwestern USA, by 3x in the Klamath, and 2x in the eastern Cascades and central and northern Rockies.

Chi-square comparisons between actual stand-age distributions at the onset of fire exclusion versus the expected stand-age distributions for a low/moderate-severity fire had exceptionally low probabilities in all regions (P<<.00001, n = 61–877). This was because plots were mostly dominated by young and intermediate aged trees prior to fire exclusion (Figs. 2A–F). The mean stand ages at the time of onset of fire exclusion were 59–114 years, depending on the region, considerably shorter than current mean ages (105–148 years: Table 5). Therefore, the FIA data were inconsistent with the hypothesis that the ponderosa pine and mixed-conifer forests of western North America, in unmanaged landscapes, were predominantly park-like with low age-class diversity due to the dominant influence of low/moderate-severity fire.

The hypothesis that mixed-severity fire prior to fire exclusion would be lower in the driest (ponderosa and Jeffrey pine) forests than other forests also was not supported. Based on stand-ages (not shown), there was as much as or more mixed-severity fire in the pine forests. In the Pacific states, we found almost identical stand-age distributions from 1800–1900 in ponderosa/Jeffrey pine stands (n = 20 plots) versus all non-ponderosa stands (n = 204 plots). Plots from the time period 1800–1900 accounted for 70% and 73%, respectively, of all plots with dominant trees that established in or before 1900. In the northern and central Rockies, 86% of ponderosa pine stands (n = 66 plots) and 81% of the non-ponderosa pine stands (n = 615 plots) that established in or before 1900 had stand-ages between 1800 and 1900 (χ^2^ = 0.85, n = 676, P>0.6). Likewise, in the southwestern USA, 98% of ponderosa pine stands (n = 96 plots) and 92% of the non-ponderosa stands (n = 37 plots) that established in or before 1900 had stand-ages between 1800 and 1900 (χ^2^ = 1.27, n = 133, P>0.25). However, when all plots were considered, significantly more stands established from 1800–1900 in ponderosa pine than non-ponderosa forests (χ^2^ = 11.96, n = 1038, P<0.001), indicating higher fire disturbance in pine forests.

### Comparing the Weight of Landscape-scale Evidence by Region

The consistency of multiple lines of evidence for mixed-severity fire in the ponderosa pine and mixed-conifer forests is an important finding. In all regions, there were tree-age data supporting considerable age-class diversity created by mixed-severity fire, and a paucity of undisturbed park-like forests. The full weight of landscape-scale evidence is greatest in the regions with area-specific rotations of severe fire from GLO data: the eastern Cascades, nearby Blue Mountains in the northern Rockies, central Rockies, and southwestern USA (Table 4). In the Cascades, these data are further supported by analyses of early aerial photography at a regional scale [35], and in small landscapes [61]–[63] and numerous historical descriptions (see [56]: Table S1). In the northern Rockies, historical documentation (e.g., [45]–[48], [50], [53], [54]) of mixed-severity regimes has been summarized in regional reviews [16], [65], [66], and stand-age reconstructions of historical fire regimes indicate mixed-severity fire in ponderosa-pine/Douglas-fir forests [67]–[69]. In the Colorado Front Range, the findings based on GLO data [57], [58] are remarkably consistent with earlier studies based on tree-ring stand reconstructions from broadly distributed samples [70]–[72]. In the southwestern USA, GLO data are supportive of mixed-severity fire on most of Black Mesa and much of the Mogollon and Coconino Plateaus [57], [59], while a number of other studies also describe evidence for mixed-severity fire [9], [14], [55], [73]–[77].

The remaining forest regions that we assessed lack GLO analyses. However, in the Sierra Nevada and Klamath regions historical surveys and early air photo data describe mixed-severity fire regimes [20], [30], [49], [51], [60], [78]–[87] (see Table S1 for descriptions). In all regions except the Klamath, there are multiple lines of evidence from landscape-scale studies, each supporting mixed-severity fire. In contrast, evidence supporting low/moderate-severity fire is confined to relatively small areas (e.g., [88]–[91]).

### Historic vs. Contemporary Fire Regimes

We did not find evidence to support the hypothesis that fire exclusion has greatly increased the prevalence of severe fire in ponderosa pine and mixed-conifer forests (Tables 4–6, and Figs. 2A–F). Comparing current versus historical high-severity fire rotations, we found that current rotations were generally longer (less high-severity fire) in the Sierra Nevada and central Rockies (Tables 4 and 6, Table S1). No direct historical comparison could be made between current and historical high-severity rotations in the Klamath and northern Rockies at the spatial scale required in Table 4, but evidence presented in Table S1 suggests that current rotations of 599 years and 500 years, respectively, may be longer. The estimated rotation of 625 years for recent high-severity fire in the southwestern USA [92] was shorter than the historical estimate of 828 years for the Mogollon Plateau in Arizona. Combining the Mogollon Plateau and Black Mesa to provide a better comparison with fire across the southwestern USA produces a historical high-severity rotation of 522 years [57]. In the eastern Cascades, high-severity fire rotations since 1984 (889 years) were longer than historical rotations (Table 6 vs. Table 4).

**Table 6:** Current high-severity fire rotations.

Region	Source	Forest Types	Time period	Rotation (yrs)
Sierra Nevada, southern Cascades	[132]	All low/mid- and mid/upper elevation conifer forests	1984–2010	645
Klamath (all)	[129]	All low/mid-elevation conifer forests	1984–2005	599
Eastern Cascades (all)	[129]	All low/mid-elevation conifer forests	1984–2005	889
Northern Rockies	[92]	Ponderosa pine forests	1980–2003	500
Central Rockies	[92]	Ponderosa pine forests	1980–2003	714
Central Rockies	[58]	Ponderosa pine forests	1984–2009	431^1^
Southwest	[92]	Ponderosa pine forests	1984–2003	625
Northwest (Eastern Cascadesand Blue Mountains)	[92]	Ponderosa pine	1984–2003	1,000

Data cited are from low/mid-elevation conifer forests in western North America.

1 Higher-severity fire: includes moderate- and high-severity.

## Discussion

### Historical Fire Regimes

The primary objective of this paper was to address how prevalent mixed-severity fire regimes were historically in ponderosa pine, mixed conifer, and other low- to mid- elevation, montane forests of western North America. We hypothesized that age-class diversity was low, consistent with long-term effects of low/moderate-severity fire regimes (Table 1). We reviewed evidence with inference across both large and smaller landscapes across many forest regions. The majority of the evidence did not support the low/moderate-severity fire hypothesis, but, instead, supported the alternate hypothesis that mixed-severity fire shaped these forest landscapes. This finding applies to Pacific states ponderosa pine, Jeffrey pine, and California mixed-conifer forests, as well as ponderosa pine and mixed-conifer forests in the eastern Cascades, Rockies and southwestern USA, where low/moderate-severity regimes have often been applied. In some areas (Blue Mountains, Mogollon and Coconino Plateaus) high-severity fire occurred at less frequent intervals (rotations of 828–849 years) [57], [59]. Even at these rotations, high-severity fire creates considerable age-class diversity in a landscape, and moderate/high-severity fire rotations were 235–319 years, which further enhances diversity (with small groupings of high-severity fire interspersed within moderate-severity fire areas).

FIA stand ages in the unmanaged forests in all regions reflect a pattern of high age-class diversity occurring prior to federal fire suppression policies and reductions in Native American burning (by the early 20^th^ century) with the arrival of settlers [20], [46], [49], [51] Natural disturbances occurred at rates that led to stands numerically dominated mainly by young and intermediate-aged trees. Disturbance processes dramatically declined following the onset of fire exclusion, suggesting fire was the primary disturbance agent [35]. However, in considering the age patterns of dominant trees in the FIA plots, it is essential to also address alternative explanations for the dominance of young- and intermediate-aged stands prior to fire exclusion, such as climate variability and disturbance by insect outbreaks.

While we recognize that climate variability influences rates of tree regeneration generally [93], and may determine success or failure of tree regeneration specifically following disturbance, we believe that the broad patterns of dominant overstory tree ages in the FIA plots mainly reflect the effects of past fire for several reasons. The dominant stand ages of young and intermediate aged trees prior to fire exclusion are consistent with periodic disturbances with significant tree mortality that shifted dominance to a new generation of trees, rather than solely episodic tree establishment due to climatic variation at a multi-decadal scale. This is supported by research in the central Rockies where, at a multi-decadal time scales, large datasets of tree recruitment dates over the past c. 250 years do not correlate with moister climate at the same time scale, but instead correlate with drier climate that was conducive to high-severity fires [70]–[72], [91]. Likewise, studies in the same area show that outbreaks of bark beetles and defoliators result in growth releases of non-host trees rather than even-aged, multi-species tree cohorts [94], [95], thus facilitating discrimination from post-fire stand structures [91]. Fire exclusion was likely effective in some areas between 1900 and 1930, which could have led to understory tree recruitment in this time frame. However, research suggests that in some areas the favorable influences of timber harvesting and/or cattle grazing on tree establishment may confound the attribution of tree recruitment to fire exclusion [96]. In addition, the plot age data demonstrate that recruitment was just as common or more common in decades before 1900 as between 1890 and 1930. Lastly, while it is possible that greater mortality in older trees, from competition or insects and pathogens, might explain high levels of recruitment prior to fire exclusion, we do not see this pattern during the suppression era. Thus, higher levels of mortality in older trees seems likely to have been caused by fire.

Our findings illustrate the need for studies with a spatial scale of inference suited to describing patterns across large, heterogeneous landscapes. This is illustrated by three recent studies from old forest stands (one in the Black Hills (500 ha), one in the Sierra Nevada (3,000 ha), and one in the southwestern USA (307 ha)) that reported very little or no historical high-severity fire, and hence low-severity regimes (Table S1: [88]–[90]). In contrast, broader-scale analyses of historical data for the Sierra Nevada (Table S1: [78]:), Black Hills [65], and southwestern USA [57] suggest fire regimes in the broader landscape within which these three studies occurred were mixed-severity.

A fourth study [97] analyzed 1914–1922 Bureau of Indian Affairs (BIA) timber cruise plot data from within a larger area (38,651 ha), and found relatively low tree densities in ponderosa pine and mixed-conifer forests of the eastern Klamath region in Oregon, and suggested that forests were too open to support any significant crown fire. However, only a subset of the townships surveyed by BIA in these forest types were included in the analysis (Table S1), and the surveys did not include trees 10–15 cm dbh, which comprise ∼20% of all trees [97], and most surveys did not include lodgepole pine, which comprise ∼10% or more of these forest types in that region within unlogged areas [49]. In addition, historical data indicate that extensive timber harvesting had occurred in the areas analyzed by 1914–1922 (Table S1), and evidence of previous timber harvesting was not among the factors that BIA surveyors were required to note (Table S1). Tree densities in unlogged reference ponderosa pine and mixed-conifer forests in this landscape from the late 19^th^ century and early 20^th^ century indicate much denser and more variable forest conditions (Table S1). Also, USGS surveys conducted in the 1890s within unlogged ponderosa pine and mixed-conifer forests across a larger expanse (310,267 ha) map substantial high-severity fire from 1855–1900 (high-severity rotation of 352 years), suggesting a mixed-severity regime (Table S1).

The absence of evidence for mixed-severity fire in some older forests selected for study may be due to fire exclusion. If the effect of fire exclusion in reducing mixed-severity fire is not accounted for in describing reference conditions, it may lead to shifting baseline syndrome (i.e., a system is not measured against the true baseline, but against one that already has departed from the true baseline [98]). This effect may be caused or compounded by diminishing evidence of age-class diversity. For example, high-severity fire can be mapped at landscape scales from early air photos [9], [17], [61]–[63], [99], but the same historic fire effects may not be visible from current imagery that can be used for assessing landscape-scale patterns.

Data with greater temporal depth than analyzed here can better capture past variability in the frequency of large fire events. Thus, it is noteworthy that paleoecological studies also support mixed-severity fire regimes for the ponderosa pine and mixed-conifer forests. These studies have found charcoal depositions from major fire episodes in ponderosa pine and interior Douglas-fir forests occurring for millennia in the northern Rockies (central Idaho: [100], [101]), Klamath [102], Sierra Nevada [103], eastern Oregon Cascades [104], and southwestern USA [105]–[107]. These major episodes are generally interpreted as large, severe fire events [101]–[107].

The occurrence of mixed-severity fire prior to fire exclusion is also well supported by another line of evidence: the potential behavior of wildfire as affected by weather and climate. Based on direct observations of fire behavior, high winds (generally 10 m open wind speeds >32–35 kilometers/hr) may subject virtually any conifer forest, regardless of fuel density, to crown fire [108]. Thus, empirical data call into question a major premise of the low/moderate-severity fire regime: that ponderosa pine and mixed-conifer forests may be completely resistant to crown fire. Fire intensity increases with winds, and at winds of >30 km/hr spot fires may be ignited over 1 km ahead of the fire front [109]. The coalescing of separate spot fires with the fire front can further energize wind-driven fire [110], [111]. Severe droughts also intensify fires by reducing fuel moisture to extremely low levels, allowing crown fire under less windy conditions [108], [112]. Severe drought years throughout much of western North America occurred from 1856 to 1865, 1870 to 1877 and 1890 to 1896 [113]. The extensive high-severity fires of 1910 (the Big Burn in Idaho and Montana), when large areas of drier forests burned at high severity prior to fire exclusion–much of it in ponderosa pine–illustrate how fire behavior that is rare temporally due to extreme climate and weather can dominate in space [1]. Many fire episodes in the charcoal records that exceed modern fires undoubtedly involve combinations of extreme wind, drought, and mass fire.

The largest patch sizes of high-severity fire likely occurred during the most extreme conditions for fire behavior. While patch sizes of high-severity fire are difficult to document, it follows from commonly observed heavy-tailed distributions of patch sizes created by fire [114], [115] that very large patches of high-severity fire (thousands of ha, e.g., [17: Fig. 1, 58]) were a primary reason why considerable area exhibited forest structure consistent with high-severity fire historically in all regions. Large patches, though numerically subordinate, are dominant in terms of total area burned, while the opposite applies to small patches [58].

There is abundant evidence that past forests may not have required extreme weather and climate for mixed-severity fire to have occurred. Younger, more flammable forests [32] appear to have been widespread in dry-forest regions based on dominant stand ages prior to fire exclusion (Fig. 2). In addition, the ranges in fire-free intervals in many low- to mid-elevation forested areas were sufficient to allow for substantial vegetation growth and recovery of fuels between fires (e.g., 20–50+ year rotations [61]–[63], [116]–[118]). For example, in the Sierra Nevada, fuels may recover to pre-burn levels in nine years [119], [120], so fire-free interludes (or fire rotations), more often than not, may have been sufficient to allow growth of significant amounts of high-energy shrub fuels. In describing low/mid-elevation forests throughout the northern Sierra Nevada, Leiberg [51: page 32) states: “There is a great amount of undergrowth in the forest which has attained its present proportions chiefly through the agency of fire. Most of it [undergrowth] consists of species of *Ceanothus*.” For mid-elevation forests, he reports (page 37): “Nearly all the type situated at altitudes below 7,000’ [2134 m] carries a vast amount of undergrowth. It consists mainly of manzanita [*Arctostaphylos* spp.], ceanothus, and scrub oak [*Quercus chrysolepis, Q.vaccinifolia*].” Similarly abundant shrub fuels were also documented historically in the westside of the central/southern Sierra Nevada [51], in the eastern Oregon Cascades [56: Appendix A] and in Oregon’s Blue Mountains [57]. Flame lengths in actively burning manzanita and ceanothus are typically 4–5 times the ∼1–2 m height of the shrubs, sufficient to cause ignition of forest canopy tree crowns under favorable burning conditions. Many of these shrub species recruit primarily, if not exclusively, after severe fire, and their occurrence is a further indication of the historical presence of such fire [121].

### Changes in Fire Regimes and Stand Age Distributions with Fire Exclusion

We also hypothesized, consistent with existing concerns about unprecedented fire severity in western North America (e.g., [6]–[9], [11], [13], [15], [17], [28]), that fire exclusion has greatly increased the prevalence of severe fire in ponderosa pine and mixed-conifer forests. We found little support for this hypothesis. Over the full period of effective fire exclusion in unmanaged forests, average ages of dominant overstory trees in FIA plots suggest there has been about a threefold to fourfold decrease in stand initiation due to fire in the Sierra Nevada, Klamath, and southwestern USA, and about a twofold decrease in the eastern Cascades, central and northern Rockies (Figs. 2A–F). In addition, patch sizes of high-severity fire in the central Rockies have not increased [58]. Our assessment of high-severity rotations based upon existing literature also revealed a generally lower incidence of high-severity fire in these forests in recent decades (Tables 4 and 6, and S1).

## Conclusion

The importance of multiple lines of evidence has been stressed in determining whether mixed-severity fire regimes applied historically [122]. Our results illustrate broad evidence of mixed-severity fire regimes in ponderosa pine and mixed-conifer forests of western North America. Prior to settlement and fire exclusion, these forests historically exhibited much greater structural and successional diversity than implied by the low/moderate-severity model (Table 2). Lack of recognition of past variability in fire may be due, in part, to common misclassifications of fire regimes. To improve clarity in communication, we propose that “low/moderate-severity” be applied to those regimes where, as the term implies, high-severity fire is absent. These circumstances appear to be quite rare in the ponderosa pine and mixed-conifer forests of western North America. Therefore, a fire regime with a high-severity component of any amount should not be classified as low/moderate-severity [e.g., 9,17,28].

Our findings suggest a need to recognize mixed-severity fire regimes (Table 2) as the predominant fire regime for most of the ponderosa pine and mixed-conifer forests of western North America. Given societal aversion to wildfires, the threat to human assets from wildfires, and anticipated effects of climate change on future wildfires, many will question the wisdom of incorporating historical mixed-severity fire into management goals. However, focusing fire risk reduction activities adjacent to homes is needed to protect communities [123], and this may expand opportunities for managed wildland fire–away from towns–for ecological benefits of fire-dependent biota. However, a major challenge lies with the transfer of information needed to move the public and decision-makers from the current perspective–that the effects of contemporary mixed-severity fire events are unnatural, harmful, inappropriate and more extensive due to fire exclusion–to embrace a different paradigm [124]. This paradigm would not emphasize a single, appropriate condition, but would explicitly recognize the vital role of variation in fire in maintaining successional diversity and fire-dependent biota [125], and allow natural rates of ecological succession [18], [19], [126]–[128]. It would also recognize that these effects have generally diminished, and that more fire, including high-severity fire, where it is in deficit, is an ecologically desirable goal. Of course, while most current research indicates that fire severity is not increasing in ponderosa pine and mixed-conifer forests of western North America [129]–[132], it will be critical to continually assess fire regimes in a changing climate.

For management, perhaps the most profound implication of this study is that the need for forest “restoration” designed to reduce variation in fire behavior may be much less extensive than implied by many current forest management plans or promoted by recent legislation. Incorporating mixed-severity fire into management goals, and adapting human communities to fire by focusing fire risk reduction activities adjacent to homes [123], may help maintain characteristic biodiversity, expand opportunities to manage fire for ecological benefits, reduce management costs, and protect human communities.

## Supporting Information

Table S1 **Evidence of historic fire severity in ponderosa pine and mixed-conifer forests of western North America.** A summary of published studies and historical documents that provide evidence regarding mixed-severity fire in the ponderosa pine and mixed-conifer forests of western North America, but do not provide sufficient information to estimate high-severity fire rotations, or were conducted in smaller landscapes. Many fire scar studies have also been done in these forests, but fire scars alone are not sufficient to distinguish low- from mixed-severity regimes.
(DOCX)
