# Supplementary material for: Examining Historical and Current Mixed-Severity Fire Regimes in Ponderosa Pine and Mixed-Conifer Forests of Western North America
Source: PLoS One. 2014 Feb 3;9(2):e87852. doi: 10.1371/journal.pone.0087852 (PMC3912150; doi:10.1371/journal.pone.0087852)
Supplement: Table S1 — Evidence of historic fire severity in ponderosa pine and mixed-conifer forests of western North America. A summary of published studies and historical documents that provide evidence regarding mixed-severity fire in the ponderosa pine and mixed-conifer forests of western North America, but do not provide sufficient information to estimate high-severity fire rotations, or were conducted in smaller landscapes. Many fire scar studies have also been done in these forests, but fire scars alone are not sufficient to distinguish low- from mixed-severity regimes. (DOCX) [file pone.0087852.s001.docx]

Dennis C. Odion, Chad T. Hanson, André Arsenault, William L. Baker, Dominick A. DellaSala, Richard L. Hutto, Walt Klenner, Max A. Moritz, Rosemary Sherriff, Thomas T. Veblen, Mark A. Williams. Examining historical and current mixed-severity fire regimes in ponderosa pine and mixed-conifer forests of western North America.

Table S1. **Evidence of historic fire severity in ponderosa pine and mixed-conifer forests of western North America**. A summary of published studies and historical documents that provide evidence regarding mixed-severity fire in the ponderosa pine and mixed-conifer forests of western North America, but do not provide sufficient information to estimate high-severity fire rotations, or were conducted in smaller landscapes. Many fire scar studies have also been done in these forests, but fire scars alone are not sufficient to distinguish low- from mixed-severity regimes.

| Location |  | Source |  | Description of high-severity fire evidence |
| --- | --- | --- | --- | --- |
| Pacific States | California | [79] | Low-mid elevation montane conifer forests | Monograph of the effects of fire on timber resources. Abundant descriptions and photos showing extensive areas of forests converted to chaparral by fire in the early 1900s, including some high-severity fire patches hundreds, and thousands, of ha in size within ponderosa pine and mixed-conifer forest. |
|  | Sierra Nevada | [82] | Lower to upper montane forests | Comprehensive report to Congress on the status of the Sierra Nevada by numerous scientists contains an “Alternative View” that “stand-threatening” fires were not rare or uncommon but were probably more frequent than commonly assumed as a result of combinations of prolonged drought, an accumulation of dead material from natural causes and severe fire weather. It further points out that “in the words of John Muir, ‘there was a mix of dark, dense, or thick forests in unknown comparative quantities’” and cites a detailed diary which recorded dense conditions six times more often than the open conditions associated with frequent surface fire. |
|  | Sierra Nevada | [20,86] | White fir, Ponderosa pine | Large amounts of chaparral in landscape were created by historical high-severity fires. |
|  | Sierra Nevada | [81] | Giant Sequoia and mixed-conifer forests | High-severity patches beneficial for Giant Sequoia reproduction. Large stand-replacing fires are not unprecedented. |
|  | Sierra Nevada | [141]^1^ | Mixed-conifer, Jeffrey pine and white fir | The current fire regime with a stand-initiating rotation of ~200 years is presumed to be similar to the historical one because fires have been allowed to burn since 1974. “While it is clear that patches were generally small (≤ 4 ha), large contiguous stand-replacing patches did occur (Figs. 2 and 4).” |
|  | Sierra Nevada (Lake Tahoe basin) | [142]^2^ | Jeffrey pine, white and red fir, mixed-conifer | Proportion of landscape burned in most recent pre-suppression fires in which 10-20 emergent trees/ha (moderate severity), or <10 emergent trees/ha (high severity), remained after fire ranged from 47-87% and 2-50% percent, respectively, depending on slope position and aspect, indicating mixed-severity fire. Rotations are indeterminable due to lack of time period for these fire effects. |
|  | Sierra Nevada | [99] | Jeffrey pine, white and red fir, mixed-conifer | The occurrence of “extensive, pre-suppression, stand replacing fire” in the landscape at the south end of Lake Tahoe was supported using historical evidence, aerial photographs, and stand-age analysis. |
|  | Sierra Nevada | [90] | Mixed-conifer, ponderosa pine, Douglas-fir, incense-cedar | Sampling at 85 points (three nested subplots, ranging from 0.01 to 0.1 ha depending upon tree size, at each point) within a 2125-ha old-growth forest area found little evidence of high-severity fire except at the scale of small groups of trees. The stand ages in study area with many dominant trees ~300 old were unusual in the Sierra Nevada (Fig. 2A). |
|  | Sierra Nevada | [143] | Mixed-conifer, ponderosa pine, Douglas-fir, incense-cedar | No clear evidence of high-severity fire found in four 0.01-ha subplots within each of 28 plots, each 1.6 ha in size, within a 4000-ha old-growth forest area selected for study. |
|  | Sierra Nevada | [78] | Mixed-conifer, ponderosa pine, Douglas-fir, incense-cedar | Surveys conducted within primary forest to evaluate timber production potential in 1.62-ha plots in 16.2-ha sub-sections within each 259.1-ha (640-acre) section on the westside of the Stanislaus National Forest, portions of which are now in Yosemite National Park and were included in the studies by [90] and [143]. Surveys for individual tree size, density and species were not conducted in areas that had recently experienced high-severity fire. Surveyors noted that the dominant vegetation cover across many 259.1-ha sections was montane chaparral and young conifer regeneration following high-severity fire. For example (from a representative township in the data set): a) T1S, R18E, Section 9 (“Severe fire went through [this section] years ago and killed most of the trees and land was reverted to brush; b) T1S, R18E, Section 14 (“Fires have killed most of timber and most of section has reverted to brush”); c) T1S, R18E, Section 15 (same); d) T1S, R18E, Section 23 (“Most of timber on section has been killed by fires which occurred many years ago”); e) T1S, R18E, Section 21 (“Old fires killed most of timber on this section and most of area is now brushland”). |
|  | S. Cascades | [61] | Mixed-conifer, Jeffrey pine, white fir, and Douglas-fir on lower slopes | From 1883-1926, 6-23% of lower and middle slopes of 1600 ha study area experienced high-severity fire, and 86% of upper slopes experienced high-severity fire. High-severity was < 10 emergent trees/ha remaining after fire. Low-severity fire ranged from 60% (lower slopes) to 1% (upper slopes). |
|  | S. Cascades | [62,63] | Mixed-conifer, Jeffrey/ponderosa pine and white fir | From 1889-1918 moderate- to high-severity fire (primarily high) was predominant in 6,000 ha study area. Low/moderate-severity fire ranged from ~35-48%, indicating mixed-severity fire. |
|  | S. Cascades | [144] | Ponderosa pine | There was no clear evidence of high-severity fire in seven 0.9-1.0 ha plots in a 235-ha old growth forest. |
|  | S. Cascades | [85] | Red and white fir | Severe fires initiated large cohorts of white fir, while more frequent low-severity fires thinned forest structure. |
|  | S. Cascades and E. Klamath | [49] | Dry Douglas fir, and mixed-conifer | “In T. 37 S., R. 5 E., occurs a growth of white fir nearly 75 per cent pure covering between 4,000 and 5,000 acres [1616-2024 ha]. It is an even-aged stand 100 years old and is clearly a reforestation after a fire which destroyed an old growth of red fir [Douglas-fir] one hundred and five or one hundred and ten years ago [i.e., prior to 1800].” |
|  | S. Cascades and E. Klamath | [49] | Mixed-conifer and ponderosa pine | Data on the extent of 75-100% timber volume mortality from 1855-1900 are presented by USGS surveyors in the late 19^th^ century. Over this time period, within forests comprised by >50% ponderosa pine in townships with no logging (310,267 ha of forest), there were 39,595 ha of high-severity fire (75-100% volume mortality) for a rotation of 352 years. |
|  | Klamath (western) | [80] | Douglas-fir, mixed-conifer | Three 5-8 ha sites studied. Even-aged stands and stands with 2 age classes were common as a result of moderately severe to severe fire at intervals of about 140 years. This patchy fire behavior created an age mosaic in the landscape. |
|  | Klamath | [60] | Douglas-fir, mixed-conifer | From 1850 to1950, 12-31% of 1600 ha study area experienced high-severity fire and 35-63 percent experienced moderate and high severity fire, depending on aspect. High-severity was <10 emergent trees/ha remaining after fire. High and moderate severity was <20 emergent trees/ha remaining after fire. Low severity fire was also common. |
|  | Klamath | [84] | Low elevation mixed-conifer pine and fir | In the Klamath Mountains and southern Cascades, large (100 ha) mainly even-aged patches of trees are present indicating that high-severity burns played an integral role in shaping forest structure at stand and landscape scales.” There was evidence of high-severity fire in several stands in Rusch Creek and in adjacent watersheds.” Stands of knobcone pine (*P. attenuata*) and montane chaparral (*Arctostaphylos* sp.;*Ceanothus* sp.) were present on some upper slopes and ridgetops.” |
|  | E. Klamath | [97] | Ponderosa pine, mixed-conifer | Using Bureau of Indian Affairs (BIA) timber cruise data from 1914-1922, densities of trees >15 cm dbh ranged from 38-88 trees/ha in three study areas in the former Klamath Indian Reservation in ponderosa pine and mixed-conifer forests—less than the ~150 trees/ha >10 cm dbh found in this area in 19^th^ century GLO surveys within these forests prior to logging [56], or the 152 trees/ha >10 cm dbh in “virgin” (unlogged) ponderosa pine forest in this area, based upon numerous “large sample plots” (totaling 64 ha) in stands “representative” of average forest conditions [140]. It was hypothesized [97] that these forests had a low-severity regime and were too open to support significant crown fire. Expanding this area of interest to a larger landscape indicates a mixed-severity fire regime with a high-severity fire rotation of 352 years [49] (see above). Differences in historic tree density estimates exemplify challenges identifying reference conditions. Many of the BIA 1914-1922 survey areas had been logged by then [49: p. 401], [145: pp. 65-66 (Chiloquin), [146: p. 12 (Trout Creek, R9E/T36S), and BIA surveys did not note logging [147]. The BIA surveys included many ponderosa pine townships not analyzed in [97] (see [147]). |
| Rockies | Northern | [66] | Ponderosa pine, Douglas-fir | Historical sources (e.g., [45-48,50,53,54] and tree-ring reconstructions document that, near or before A.D. 1900, the low-severity model may apply in dry, low-elevation settings, but that fires naturally varied in severity in most forests. Low-severity fires were common, but high-severity fires also burned commonly, sometimes covering thousands of hectares. |
|  | Northern (southern interior B.C.) | [16] | Dry ponderosa pine | Four sources of information were used to infer historical disturbance regimes: (1) patterns of annual and seasonal weather and lightning strikes, (2) topographic variability, (3) records of wildfire, insect attack, and timber harvesting practices, and (4) early systematic forest surveys. Historic natural disturbances were likely diverse and episodic at multiple spatial and temporal scales, consistent with a mixed-severity disturbance regime. |
|  |  | [69] | Dry ponderosa pine | A stand-age analysis was conducted in a 1,105-ha study area, using 41 plots 2-ha in size each. Fire regimes were dominated by mixed/high-severity fire. |
|  | Northern (Montana) | [38] | Dry ponderosa pine/Douglas-fir | Mixed-severity fire regimes found, using stand-age analyses of nine 1-ha plots, with inferred high-severity fire rotations of 150-400 years. |
|  | Northern (Idaho) | [68] | Dry ponderosa pine/Douglas-fir | Mixed-severity fire regimes inferred with stand-age analysis, based on transects through two study areas (1,200 and 1,600 ha), with inferred high-severity fire rotations of 40-200 years. |
|  | Central (Colorado Front Range) | [148] | Primarily ponderosa pine/Douglas-fir | Sixty-eight landscape photographs taken between 1870 and 1915 and repeated in 1984-85 show high severity burning of forests dominated by ponderosa pine and Douglas-fir over areas > 100s of hectares. Tree ages indicated that, following these high severity fires in the 19^th^ century, abundant tree establishment has resulted in even-aged cohorts. |
|  | Central (Colorado Front Range) | [149] | Ponderosa pine | All stands dominated by trees that established prior to c. 1900 A.D. originated after high-severity fires occurring in the 18^th^ to mid-19^th^ centuries that triggered major pulses of tree establishment resulting in even-aged cohorts. |
|  | Central (Colorado Front Range) | [72] | Primarily ponderosa pine and Douglas-fir | In the upper montane zone where ponderosa pine is the dominant forest type across half the forest area, of the 40 sites sampled, 62% historically experienced predominantly moderate-severity fire, 38% burned at high severity, and no sites burned exclusively at low severity. Most mature trees in the current forest established during a multi-decadal period of extreme drought and fire from 1850 to 1889. |
|  | Central (Colorado Front Range) | [58] | Primarily Ponderosa pine and Douglas-fir | Higher severity burn patches, size distribution, and fire rotation for the 1800s (A.D. 1809–1883) were reconstructed using General Land Office (GLO) survey transect data and compared to the characteristics of modern fires over a recent 26-year period (A.D. 1984–2009) taken from remotely sensed data. The historical geometric mean higher severity patch was 170.9 ha with a maximum size of 8,331 ha. The current geometric mean was 90 ha and a maximum size of 5,183 ha. |
|  | Central (Colorado Front Range) | [70] | Primarily ponderosa pine | Over the elevation range of ponderosa pine from 1800 to 2800 m, evidence of low severity historical fire (primarily trees with multiple fire scars) indicates that low severity fires occurred mainly below 1950 m. In contrast, above 2200 m in current dense forests, only 2 to 52% of trees in 18 stands pre-date the last moderate to high-severity fires occurring mostly in the late 19^th^ century, thus indicating that fire severities prior to any fire exclusion effects was sufficient to kill high percentages of mature trees. |
|  | Central (Colorado Front Range) | [71] | Primarily ponderosa pine | Across the elevation range of ponderosa pine in the northern Front Range, spatially comprehensive reconstruction of historical fire regimes from fire scars and tree ages indicates that > 80% of the zone was characterized by infrequent fire (intervals > 30 years) associated with moderate and high fire severities. Less than 20% of the ponderosa pine zone was characterized by relatively frequent (return intervals < 30 years) and low severity fires. Of 54 sites sampled to test the validity of the spatial reconstruction, 81% showed mixed- and high-severity fire effects, rather than low-severity effects, prior to fire suppression. |
|  | Central (Colorado Front Range) | [117] | Ponderosa pine/Douglas-fir | In a 4000 ha study area fire severity varied from low-severity surface fires to large stand-replacing fires. “Large portions of the landscape did not record any fire for a 129 year-long period from 1723 to 1851.” “Large fires with significant areas of complete overstory tree mortality were a component of the fire regime in ponderosa pine forests at Cheesman Lake.” |
|  | Central (Colorado Front Range) | [150] | Ponderosa pine/Douglas-fir | Three quarters or more of the 35 km^2^ unlogged landscape have distinct tree age gaps with all trees post-dating fire-scar dates indicating occurrence of stand-replacing fires. Most fires apparently included both a surface and stand-replacing component but “there is no evidence of frequent surface fires in any one location (e.g. multiple fire scars a few years apart on single trees) as is typical in the ponderosa pine/bunch grass system in the southwestern United States.” |
|  | Wyoming and South Dakota | [65] | Ponderosa pine | Historical sources (e.g., Graves 1899) indicate that several large stand-replacing fires occurred between 1730 and 1852. |
|  | South Dakota | [77] | Ponderosa pine | The fire scar record extends from 1529-1893 and 3.3 percent of fire was crown fire during this period. The 500-ha study area was located in old-growth ponderosa pine, portions of which were logged and grazed. |
| Southwest | Arizona | [75] | Ponderosa pine and Douglas-fir | Stand-replacing fire occurred over 61 ha of 310 ha study area in 1867. There was evidence for four other stand-replacing events from 1775-1819. Large expanses of manzanita chaparral flanked the 310 ha study area, which could be a byproduct of severe fire historically."Multiple lines of evidence indicate that the 1867 fire was both a surface and a stand-replacing event that killed most trees within a 60 ha patch." "The fire corridor between the north and south areas was altered by a stand-replacing fire in either 1763 or 1775 that converted pine forests to shrub fields, which impeded widespread fires in subsequent years." |
|  | Arizona | [73] | Mixed conifer | "Vast denuded areas, charred stubs and fallen trunks and the general prevalence of blackened poles seem to indicate their (fires) frequency and severity...." "The old fires extended over large areas at higher altitudes (in the mixed conifer and subalpine forests) accounting for several square miles on either side of Big Park and numerous smaller irregular areas over the remainder of the forest." |
|  | Arizona | [74] | Mixed conifer | Mixed-severity fire regime. |
|  | Arizona | [59] | Ponderosa pine and piñon/juniper | Thirty-nine percent of a landscape considered to be shaped by only low-severity fire was found to be shaped by mixed-severity fire. |
|  | Arizona | [55] | Mixed conifer, ponderosa pine and piñon/juniper | Non-timbered “parks” were common in ponderosa pine belt. Observations of fire effects in piñon/juniper suggest that fire caused the same type of openings in the ponderosa pine forests and that these openings are slow to reforest in the semi-arid region. Leiberg et al. state: “The fires which burned during Indian occupancy and soon after the arrival of the present settlers in the region were far more- widespread and destructive than those of recent years [late 1800s].” This may explain why the non-forested parks they observed had not been recently created. The reason for the less severe fire was that intensive livestock grazing had removed much of the grass growth, which Leiberg et al. described as luxuriant. |
|  | Colorado | [14] | Ponderosa pine | "We suspect that the late 14th and 15th centuries were periods in which some southwestern ponderosa pine forests were more vulnerable to altered fire behavior including large crown fires." |

^1^The current fire regime in the 15 km^2^ study is presumed to be similar to the historical one because fires have been allowed to burn since 1974.

^2^A 20 km^2^ study area located in remnant, unlogged old-growth.

**Additional References**

141. Collins BM, Stephens SL (2010) Stand-replacing patches within a mixed severity fire regime: quantitative characterization using recent fires in a long-established natural fire area. Landscape Ecology 25: 927-939.

142. Beaty RM, Taylor AH (2007) Fire disturbance and forest structure in old-growth mixed conifer forests in the northern Sierra Nevada, California. Journal of Vegetation Science 18: 879-890.

143. Collins BM, Everett RG, Stephens SL (2011) Impacts of fire exclusion and recent managed fire on forest structure in old growth Sierra Nevada mixed-conifer forests. Ecosphere 2: Article 51.

144. Taylor AH (2010) Fire disturbance and forest structure in an old-growth *Pinus ponderosa* forest, southern Cascades, USA. Journal of Vegetation Science 21: 561–572.

145. Tonsfeldt W (2002) Selling Klamath Reservation timber 1910-1935. Journal of the Shaw Historical Library 16: 63-73.

146. Helfrich D (1974) Lumbering around Yainax. Klamath Echoes Number 12, pp. 11-12.

147. NARA (1914-1922) Timber Inventory Tally Sheets. Records of the Bureau of Indian Affairs, Record Group 75. National Archives and Records Administration (NARA), Seattle, Washington.

148. Veblen TT, Lorenz DC (1986) Anthropogenic disturbance and recovery patterns in montane forests, Colorado Front Range. Physical Geography 7:1-24.

149. Mast JN, Veblen TT, Linhart YB (1998). Disturbance and climatic influences on age structure of ponderosa pine at the pine/grassland ecotone, Colorado Front Range. Journal of Biogeography 25:743-755.

150. Kaufmann MR, Regan CM, Brown PM (2000) Heterogeneity in ponderosa pine/Douglas-fir forests: age and size structure in unlogged and logged landscapes of central Colorado. Canadian Journal of Forest Research 30: 698-711.
